# Ethico-Political aspects of clinical judgment in opportunistic screening for cognitive impairment: Arendtian and aristotelian perspectives

**DOI:** 10.1007/s11019-022-10095-y

**Published:** 2022-06-10

**Authors:** Martin Gunnarson, Kristin Zeiler

**Affiliations:** 1 Centre for Studies in Practical Knowledge, School of Culture and Learning, Södertörn University, Huddinge, Sweden; 2 Technology and Social Change, Centre for Medical Humanities and Bioethics, Department of Thematic Studies, Linköping University, Linköping, Sweden

**Keywords:** Opportunistic screening, Clinical judgment, Hannah Arendt, Aristotle

## Abstract

This article examines a population-based opportunistic screening practice for cognitive impairment that takes place at a hospital in Sweden. At the hospital, there is a routine in place that stipulates that all patients over the age of 65 who are admitted to the ward will be offered testing for cognitive impairment, unless they have been tested within the last six months or have been diagnosed with any form of cognitive impairment. However, our analysis shows that this routine is not universally and mechanically applied. Rather, the health care professionals have developed and use an ethico-political judgment, concerning, for example, *whom* to test, *when* to offer the tests, and *how* to explain and perform them. This article explores the role and practice of this form of judgment, emphasising its political and ethical nature. The analysis is based on qualitative interviews with professionals and patients, and draws on the theories of Aristotle and Hannah Arendt.

## Introduction

“Opportunistic screening” refers to a type of screening offered to a predefined population when the opportunity arises, for example when members of the population are admitted to hospital for other reasons than that which the screening concerns. As such, it can be contrasted to population-based systematic screening, in which everyone in a predefined population is offered testing (Gunnarson et al. 2021). This article examines a population-based opportunistic screening practice for cognitive impairment that takes place at a hospital in Sweden.^1^ The practice was introduced in 2017 and implemented at the geriatric ward in the form of a written routine that stipulates that all patients over the age of 65 admitted to the ward will be offered testing for cognitive impairment, unless they have been tested within the last six months or have been diagnosed with any form of cognitive impairment. The tests used are the Mini-Mental State Examination (MMSE) and the Clock-Drawing Test (CDT). The MMSE includes tests of orientation, attention, language and memory, while the CDT tests cognitive domains such as language, executive function and visuospatial abilities. Taken together, the two tests should take about 20 min to perform. As a rule, they are performed in a separate room at the ward, but sometimes at the patient’s bedside. The tests are accompanied by detailed and standardized instructions, which, for example, specify the exact formulations the test administrator should use, and prohibit any feedback from being given to the test subject during the tests, all to ensure the reliability and uniformity of the results (Palmqvist et al. 2011).

Neither the MMSE nor the CDT are diagnostic. Further examinations are always needed in order to identify the underlying disease. It is, however, a goal of the screening practice at the studied hospital to detect cases in which further, diagnostic testing for dementia and other forms of cognitive impairment is warranted, thereby making it possible to offer adequate help and support for those affected.^2^ In this way, opportunistic screening for cognitive impairment can function as the first step of a series of tests that eventually lead to a diagnosis.

Offering screening tests for cognitive impairment to patients hospitalized for other reasons is not uncontroversial. One reason for this is that the screening instruments cannot clearly distinguish between different medical conditions, for example, delirium and cognitive impairment.^3^ This may lead to a high number of false positives, commentators argue, causing unnecessary concern among patients (Burn et al. 2018; Jackson et al. 2013).

This article is based on analyses of qualitative interviews with health care professionals who work with the opportunistic screening routine at the studied hospital, and patients who have undergone the tests there. The interviews led us to realize that the screening routine is not universally and mechanically applied, but that the professionals have developed and use a form of careful judgment, concerning, for example, *whom* to test, *when* to offer the tests, and *how* to explain and perform them. The aim of the article is to explore the role of this judgment – hereafter termed “clinical judgment”. Our main focus is on the health care professionals. We examine why the professionals deem it necessary to develop and use such judgment, what it consists of, and how they exercise it. The patient interviews play a more marginal role in the article, but they are nevertheless important since they indicate how the practice of clinical judgment was experienced on the receiving end of the test relationship.^4^

## Clinical Judgment in Medicine and Screening Practices

The presence of professional judgment in medicine has been given considerable attention during the past few decades, most often conceptualized as “clinical judgment”. To put it simply, clinical judgment is understood to be the kind of rationality that enables medical practitioners to decide what to do in relation to a particular patient. The scientific and technical dimensions of medical knowing are not sufficient in this regard, since they deal with universals. Another capacity is needed, one that is sensitive to the particulars and the way they relate to the universals. In the vast literature on clinical judgment, the concept has been theorized in multiple ways: as “tacit knowledge,” “reflection in action,” and “gestalt cognition” (Kienle and Kiene 2010), as “skilled coping” (Thornton 2010), as “prudence” (Pellegrino and Thomasma 1993), as a “cognitive process” that results in a “holistic assessment of a patient’s situation” (Manetti 2019), as “artistry” (Paterson et al. 2005), and so on. Much of this research also engages with Aristoteles’s term *phronesis* (see e.g. Montgomery 2006; Shutzberg 2020), and examines the ethical dimensions of clinical judgment. We follow here this last line of theorization, using Aristotle’s term *phronesis*, which enables us to explore the ethical aspects of clinical judgment and how these are tied to situational circumstances, practical knowledge, and professional experience. However, we also use insights from Hannah Arendt’s theory of judgment, which allows us to explore what we – along the line of Arendt’s reasoning – understand as a political dimension of clinical judgement. This has received much less attention in the literature.

The most common form of screening is population-based systematic screening, and clinical judgment is rarely, if ever, mentioned in connection with such screening.^5^ This can largely be attributed to the formalized and systematized nature of this type of screening. It is typically offered to everyone within a predefined population, and performed at pre-determined time intervals. Thus, clinicians’ judgments about whom, when, and how to test have little or no place here. This echoes the conclusions drawn by Ranson et al. (2018) and Mate et al. (2017), for whom clinical judgment is the distinguishing feature between screening and what has been termed “case-finding”.

On paper, this seems to apply also to the opportunistic screening practice that is applied at the studied hospital; the routine states that everyone who belongs to a predefined group should be offered testing when they attend the geriatric ward. Two previous publications, however, suggest that more is at stake. In a study close to the theme of this article, Swallow (2016) found that the use of cognitive testing tools in clinical practice requires clinical judgment. This is so, she argues, because the circumstances of the practice shape how the tools are understood and how they can be used. In their study of the implementation of a screening tool for the detection of postpartum depression, Ben-David et al. (2017) made similar findings. They found that the clinical judgment of the nurses responsible for performing the tests often took the form of situationally and contextually informed departures from the standardized test protocol. As we will see below, we make similar findings. However, neither Swallow nor Ben-David et al. address the ethical and political dimensions of clinical judgment, which, we will try to show, are essential dimensions of this form of judgment.

## An Ethico-Political Form of Clinical Judgment

If one looks only at the written routine for the opportunistic screening practice implemented at the studied hospital, it seems to be enough to learn how to apply the pre-established routines and procedures to be able to perform the cognitive testing. In Aristotelian terms, this means that the knowledge required to offer and perform the tests is limited to *techne*. *Techne* is, according to Aristotle, a form of practical knowledge that relates to the instrumental and productive side of human activities (*poiesis*) (Aristotle 2011, 119 [1140a]). *Techne* is what we need when we set out to make something. The something (*ergon*) that we want to make lies outside the scope of the knowledge (*techne*) required to make it. For example, we do not need to know why a chair is made in order to be able to make it. On paper, the same is true for the studied opportunistic screening practice. In order to be able to offer and administer the tests, one merely has to learn the *tech*nical skills involved in translating the routine and test manuals into practice. One does not have to deliberate about the objectives of the practice. These objectives are established from the outset, and, although they are what the practice aims to realize (its *ergon*), they have nothing to do with the skills involved in performing the tests. But this reasoning is valid only on paper. In reality, as we learnt in our interviews, the *tech*nical knowledge is not sufficient. The test situations also require an ability to judge what it is right to do in a particular situation and in relation to a particular patient.

Aristotle describes this ability to judge as another form of practical knowledge, one that he terms *phronesis*. If *techne* is knowing how to make something, *phronesis* is knowing how to act. And the goal of action is not to make something external to that action, but to act well (Aristotle 2011, 120 [1140b]). In order to act well, the action must be guided by some notion of what is good. This is where Aristotle’s virtues of character come in.^6^ These virtues provide guidance about what to aim at in action.^7^ They are, however, themselves intimately tied up with action. The virtue of courage, for example, cannot be ascribed to a person who does not *act* courageously. Moreover, actions can only be performed in particular situations; there are no universal situations in which one can act (cf. Aristotle 2011, 118 [1139b]). Consequently, the virtues of character cannot be understood as immutable universals that are simply applied to a particular situation. What is a courageous act depends on the situation, and thus the definition of courage cannot be settled once and for all (cf. Gadamer 2013, 331). In Aristotle’s view, *phronesis* is what enables us to act despite varying situational circumstances, since it is the form of knowledge, or intellectual virtue, that enables us to judge what constitutes virtuous action in a particular situation, and to realize that judgment through action. Thus, *phronesis* and the virtues of character are mutually dependent (Aristotle 2011, 117 [1139a-b]).

*Phronesis* is, furthermore, intimately tied up with the acting person. Aristotle claims that a person who acts virtuously cultivates her character, and in this way strives towards a good life (*eudamonia*). This entails connecting the particular and the universal, to the extent that it is only through particular, virtuous acts that the good life in general can be achieved. *Phronesis*, Aristotle states, “is not concerned with the universals alone but must also be acquainted with the particulars: it is bound up with action, and action concerns the particulars” (Aristotle 2011, 124 [1141b]). This leads to the conclusion that experience might be more important than knowledge of universals. *Phronesis* is typically more common among older, more experienced people, Aristotle observes (Aristotle 2011, 125 [1141b]). This tells us that it does not disappear when the situation in which one has acted ends: it sediments into one’s character. Conversely, one’s character and one’s pursuit of the good life are what guides action in a particular situation. But the virtues can only ever guide: they cannot determine once and for all the right action in a given situation. What is needed, in addition to experience, is therefore a particular attitude of the person towards herself. The acting person can neither act simply on the basis of previous experience nor let herself be blinded by passion. As Gadamer puts it: “the opposite of seeing what is right is not error or deception but blindness” (Gadamer 2013, 332). Thus, deliberation is never redundant when taking *phronetic* action. Rather, *phronesis* is the very ability to deliberate well, decide what to do, and act according to this decision (Aristotle 2011:124 [1141b]; see also Gadamer 2013, 331).

Consequently, *phronesis* can be understood as a form of judgment (MacIntyre 2011, 180; Bornemark 2017; Schwarz and Hjertström Lappalainen 2020). It is an ability to judge, with the guidance of a set of desired virtues, how one should act in the situations of which one’s life consists.

Aristotle’s account of judgment, in the form of *phronesis*, emphasises its ethical character, while Arendt’s account stresses its political nature.^8^ Just as Aristotle, however, Arendt understands judgment as something that “deals with particulars” (Arendt 1992, 13; see also Passerin d’Entrèves 2006, 380).^9^ Arendt’s main source of inspiration for her thoughts on judgment was Kant and his writings on taste in the *Critique of Judgement*.^10^ From Kant, she takes the insight that there are judgments for which there is no universal rule under which to subsume the particular. Instead, we are faced with a particular from which some kind of “general” must be “derived”. This is, in Kant’s terminology, “reflective judgment”, as opposed to “determinate judgment”, which only applies in situations in which there exists a definite universal rule from which the particular can be derived (Arendt 1992, 83; 2003, 138). Arendt considers “political judgment” to be a form of reflective judgment, and “imagination” and “common sense” to be central to it (Arendt 1992, 66). Just as Aristotle, Arendt emphasizes the need to create a certain distance from the immediacy of the particular that is to be judged. Such a distance can be achieved by imagination, she argues. Again inspired by Kant, Arendt defines imagination as “the faculty of having present what is absent” (Arendt 1992, 66). She suggests that imagination enables us to engage with the experienced particular as a representation, as something that still affects us but that is no longer immediately present for us.

Further, she holds, political judgment is primarily a “mental activity” (Arendt 1971, 69). Judgment and action are deeply intertwined, but do not coincide.^11^ Action, for Arendt, is the ability to begin something new, to set something new in motion (Arendt 1998, 177). Judgment merely guides action. As such, it opens up a space in which particular actors and actions appear in a particular light, such that a choice of how to act and an evaluation of one’s own actions and those of others becomes possible. Thus, we constantly oscillate between the two, mutually dependent, poles of judgment and action (Arendt 1971, 95). As Arendt puts it, the “judging mind removes itself only temporarily [from the world of action] and with the intention of a later return” (Arendt 1971, 92).

Imagination makes it possible for us to achieve a reflective distance from the particular to be judged, but it does not accomplish this alone. “Common sense” is also an essential ingredient. Once again, Arendt was inspired by Kant who understood common sense to be a “community sense”, without which judgments would neither be possible nor meaningful. At the root of all reflective judgments is a sense of being part of a community with whom we want to share our judgments and whose assent we seek (Arendt 1992, 72; 2003, 140). Exercising judgment would be futile without the presence of others. But the reverse is also true, without reflective judgments the world would not be common in the sense we know: it would not open up in a way that makes actors and actions appear in a particular light, making them possible to discuss and debate (Zerilli 2006, 179). An impulse to communicate is an integral part of judgment; we want to persuade others about the appropriateness of our judgments. This also means, however, that we can be held responsible for them at any time, and be required to respond to questions about them (Arendt 1992, 41).

Here we begin to see what Arendt has in mind when she describes reflective judgment as being political in nature. First, it is political, in her sense of this term, since it enables us to act and orient ourselves “in the public realm, in the common world” (Arendt 2006, 218). Second, it is political because it enables us to see beyond our “subjective private conditions”, and see things from the perspective of others (Arendt 2006, 217). This latter point is an important one that we have not discussed yet, and one that concerns the very exercise of reflective judgment. In order to make a judgment, Arendt argues, we must put ourselves in the position of the others in our community: we must imagine what their views on the particular are and take these into account when we make our own judgment. It is from this process that judgment “derives its specific validity”, its generality, as it were (Arendt 2006, 217). By taking into account the perspectives of others, political judgment gains a “relative impartiality” or “disinterestedness”, Arendt writes, by which the judging subject may distance herself from her “individual limitations”, and turn her attention towards the world (Arendt 1992, 73; 2006, 217). In the context of our study, these are essential dimensions of the clinical judgment that is exercised on the geriatric ward, because they help us see that the opportunistic testing is not performed in a sociocultural vacuum. Rather, it takes place in a politically charged context in which pervasive conceptions about what the testing is for and what a life with cognitive impairment and dementia is like already exist. As we shall see, this greatly affects how some patients experience the tests.

To sum up, combining Aristotle’s and Arendt’s theories of judgment allows us to highlight and explore what we call the ethico-political nature of clinical judgment. Although there is considerable overlap between their theories, their respective emphasis allows us to go deeper than would otherwise be possible into each of the two aspects of judgment – the ethical and the political – and to view them together. Aristotle not only emphasizes the ethical nature of judgment, which consists in relating the particular to the virtues of character one aims at in order to choose the right course of action, he also helps us understand the role played by experience and the process of learning that is involved in exercising judgment. Arendt, on the other hand, emphasizes the political dimension, in the sense of judging the particular from the perspective of the common world by taking into account the perspectives of the others involved in and/or affected by the particular. In doing so, she helps us explore the ways in which the opportunistic testing practice takes place within, and is affected by, a political realm. Aristotle and Arendt differ when it comes to the relationship between judgment and action. However, combining the two theories allows us to explore the the close relationship between deliberation and action, found in Aristotle, together with the novelty and uncertainty that characterizes judgment-guided action, found in Arendt. As our analysis shows, the health care professionals are involved in a practice in which they must act. Although these actions are carefully judged, their outcomes can never be predicted fully.

## Methods and Research Ethics

The study involved interviews with patients and health care personnel. The first author performed nine interviews with patients and the second author performed seven interviews with health care personnel (five occupational therapists and two doctors) between November 2019 and February 2020. Inclusion criteria for health care personnel were that they had performed the tests or had been responsible for the discharge procedure from the ward and that they had talked to patients who had taken the tests about their test results. Inclusion criteria for patients were that they had taken both tests and that they had received an MMSE test result of 21 or above. The personnel at the hospital considered that this test result showed that the patient in question could understand what the project was about and consent to an interview. All interviewees were informed about the project, in text and orally. They were informed that they could interrupt the interview at any time without giving a reason. Written informed consent was obtained before every interview. The study follows the research ethical principles of the Declaration of Helsinki (2008) and Swedish research ethical regulations (Etikprövningsnämnden, the Swedish Research Ethics Board: approved, Ref. No. 2019–03034).^12^

The interviews were qualitative in nature and had a semi-structured design, which meant that they were based on interview guides with certain foci, while interviewees were allowed to bring up issues they considered to be important and to expand on issues that they wanted to talk about. The interview guide for the health care personnel focused on their narrations of how the practice was performed, and their views and experiences of offering, giving information about, and assessing the tests. The interviews with patients focused on their narrations and experiences of being offered and taking the test, the thoughts and feelings that undergoing the test had aroused in them, and what the test meant for them. All interviews were audio recorded, transcribed verbatim, and pseudonymized. All names used in this article are fictitious. The authors performed a thematic analysis: the data were coded, codes were put together into broader units of analysis such as sub-themes, and sub-themes were put together into themes (Braun and Clarke 2006), based on the aims of the project. One of the themes was clinical judgment, i.e. the focus of this article.

## Why Judgment is needed

Some of the occupational therapists and doctors were initially hesitant when faced with the prospect of performing tests for cognitive impairment as part of their work at the geriatric ward. “[From the outset] we opposed it a little bit,” occupational therapist Liv says, “from an occupational therapist point of view, we were of the opinion that, no, you should perform cognitive tests such as these in the right environment, and that’s in the patient’s home environment”. Similarly, doctor Sandra was “pretty hesitant” at first, because according to the dominant medical view “One should not perform cognitive examinations on the patient during inpatient care, since they are confused and in a different environment, and all that”.

When the prospect of cognitive testing was presented to them, the occupational therapists and doctors were faced with the question of how to incorporate this new task into their professional practice. From an Aristotelian perspective, this is not just a *tech*nical issue, but also a *phronetic* one. For the professionals, it was not only a question of whether it was *possible* to incorporate the testing into their practice, but also whether it was *right* to do so. In order to make such a judgment, they had to view this new task in the context of their profession as a whole. As we saw earlier, *phronesis* does just that, it connects the particular and universal, such that particular actions are guided by a general idea of what is good. This general idea of what is good is, in turn, shaped and cultivated through particular actions.

The professionals needed to gain practical knowledge of working with the tests before they could judge whether it would be right to integrate the testing into their profession. As Aristotle states, judgment in the form of *phronesis* tends to grow with experience: it is a form knowledge that one gains by engaging in action. This was evident in our interviews.

For the occupational therapists, several experiences enabled them to make the judgment that it was possible to align the testing with their profession. These included the observation that most patients were positive to taking the test, that they had the opportunity to consult each other as colleagues, that they could simultaneously find support in and go beyond the written routine, and that the testing could be beneficial for the patient.

However, making this form of judgment also involves considering the wider societal context. No professional judgments can be made in a sociopolitical vacuum. Rather, as Arendt contends, our ability to judge is what puts us into contact with our community. This is evident on numerous occasions in our interview material. Occupational therapist Emelie, for example, argues that they are able to help people.

who perhaps would’ve sat at home not being able to manage their situation if they hadn’t been here and done the tests, actually. Because perhaps they don’t go out. They just happen to come here now. Perhaps they are not out in the community at all […]. And that’s what I feel is important, that you can capture those persons who would not themselves seek or dare to seek contact. Maybe they don’t even realize that they have these problems. They think it works fine when it is actually chaotic, maybe.

Emelie’s judgment about the value of the testing practice is intimately intertwined with a particular societal situation, a situation in which many old people live alone and have scarce social contacts, which prevents them from seeking the help they need or even noticing that they have a problem at all. The judgment Emelie makes here is political, in Arendt’s sense of the term, since she uses her imagination and community sense to see the matter from the perspective of others and her own practice and actions as part of a wider community. In making this political judgment, Emelie is able to articulate her standpoint, a standpoint that may help to alter the political realm in which she acts.

If we turn now to the clinical judgments that are made within, rather than about, the testing practice, it is clear that one of the main reasons that the *tech*nical application of the written routine is insufficient is that the practice involves the “presence of others […] whose perspectives it must take into consideration”, as Arendt puts it (Arendt 2006, 217). The professionals we interviewed strongly pointed out that each patient they meet have particular circumstances, needs, and wishes, and that they must take these particularities into account in the testing practice.

Sometimes the occupational therapists’ deliberations about such particularities lead them to make the judgment that they should not apply the written routine at all, that is, they decide that it is inappropriate to even offer the test to a particular patient. In addition to the written routine, the personnel working with the tests have developed less formal routines about whom to include and not include in the testing. For example, patients with hearing or visual impairments and patients with terminal or late-stage cancer are often excluded directly. Although less formal, these routines seem to be more *tech*nical than *phronetic*. This does not mean that the patients who do not satisfy such informal exclusion criteria are automatically offered the test. A clinical judgment must always be made, based on the circumstances of each patient.

Klara, for example, emphasizes the importance of “looking at the patient’s whole situation” and asking oneself “Is it appropriate or not?”. She often consults her colleagues when she finds making such judgments difficult, and discusses with them how to interpret a particular patient’s situation. “Even though the test should be performed according to a certain structure, there are so many other factors here”, she says and continues, “Is the patient able to sit long enough? Is she or he in pain? Has she or he recently received medication? So many things can have an impact. And sometimes perhaps there isn’t a single occasion during the inpatient care that is perfect, and then you have to be able to judge, I think”.

Some patients commented in the interviews that they had noted that the written routine was not followed slavishly, and that clinical judgments about who should be tested were made. On the one hand, they understood why such a selection was made – they had observed that several of their fellow patients already displayed clear symptoms of cognitive impairment. On the other hand, it raised some concerns, since they realized that they might not have been selected through a mechanical and universal application of the routine, but through an assessment about their suitability for undergoing the test. Patient Lars, for example, wondered whether he was “getting old and that’s why they did these tests, […] maybe they only do the tests on those who, who they suspect have something…”. Here we see an unintended effect of the co-existence of the written routine and clinical judgment, one that the professionals seemed to be unaware of.

As Klara implied in her account above, the professionals must answer not only the question of *whether* the test should be performed, but also the question of *when*. Nora agrees, stating that one has to take into account “how the patients feel and if they get nervous – this isn’t their home environment – and there’s a lot of other things to consider. [The patient may say] ‘The doctor came by earlier and told me this’. It is not always the right situation to perform the test in. […] You have to try to do it at the right time, when the patient is in as good a condition as possible.”

Here, we see clearly how an ethico-political judgment is being made, taking as its point of departure the particularities of a patient, thinking and deliberating from the perspective of this patient, while at the same time consulting one’s professional experiences and those of one’s colleagues.

As Nora’s words above imply, exercising one’s clinical judgment in the testing practice involves considering also its contextual dimensions. Two contexts seem to be of particular importance for the studied practice: the hospital environment and the wider sociocultural setting, where certain conceptions of ageing, cognition, and dementia are held. These contextual dimensions contribute significantly to the professionals’ view that the written routine is insufficient, and that clinical judgment is needed. Whereas the aim of written manuals and routines is often to reduce the effects of context, in order to produce a consistent, transparent and auditable practice (Hall 2013), the use of judgment puts the actor into contact with the contexts and enables her or him to act on and within them.

We have already seen that the hospital environment and the presence of the patients in this environment as patients create the need for judgment about *when* to offer and perform the tests. However, in the professionals’ view, the hospital setting also makes it necessary to make careful judgments about *how* to perform the tests and interpret the results. The questions concerning temporal and spatial orientation are particularly difficult for hospitalized patients, since hospitalization itself may cause temporal and spatial disorientation. This is how Emelie puts it:

We almost always get stuck on the same questions. The date, [for example]; it doesn’t feel that strange that you don’t keep track of it when you’re at the hospital. But when you’re at home, a lot of them tell me that: “When I’m at home I read the newspapers and everything and keep up with things in another way”. So the date can be difficult sometimes. Then there’s also a question about which floor we’re on, [which] is not so easy for a patient who came with the ambulance through the emergency unit, to then go up to a ward and then perhaps even change wards. And it’s not they themselves who press the buttons [in the elevator]. So it’s not so easy for them to know that. So that question can feel a little bit unfair, I think…

Emelie feels that simply following the written routine would be “unfair”, due to the contextual circumstances. Judgments must be made both about how much weight one should attach to some of the questions when evaluating the results and about how to meet the emotional reactions of patients who struggle with the questions.

Moving on now to the other contextual dimension that affects profoundly the practice: the sociocultural context. All of the interviewed professionals describe patients who, when given the opportunity to take the tests, react strongly because they associate them with dementia. The professionals describe patients who ask if they are going to be locked up, or if the purpose of the testing is to catch them out. The reactions occupational therapist Klara have met include: “‘Why should I do this’, ‘Do you think I need it?’, and ‘Do you think I’m stupid?’”. We met similar reactions in some of our interviews with patients. Lars said that he understood the tests as “a crash course in senile dementia”. Ove also associated the tests with dementia, a condition he was “terrified” of, he said. Doctor Karin elaborates on why some patients react like this:

I think it is like it was, you know, with cancer in the past. If you said “cancer”, well, then you were dead. […] And I think that it’s similar, people talk about senile dementia and that has such bad connotations […]. It becomes a term of abuse, […] and then you don’t want to get that diagnosis and this is what makes people so concerned, as I said before […]. But I think that what you need […], you need to look at it like you get the diagnosis and then there is the possibility to get help.

Here we see again how the testing practice is not immune to the context in which it takes place. The context is present in the room where the tests are performed, and deeply affects those involved. In this case we are dealing with a sociocultural context in which cognitive tests of this sort are associated with dementia, and where being diagnosed with dementia is seen as a disaster. Numerous researchers have noted that such an understanding of dementia is widespread in Sweden and elsewhere (see e.g. Clarke 2006; Hellström 2014; Van Gorp and Vergruysse 2012). As Lisa Folkmarson Käll has observed: “Western culture and popular discourse is dominated by the terrifying notion that conditions of dementia lead to an unrelenting dissolution or loss of self and identity, a mental death before physical death” (Folkmarson Käll 2017, 359).

As the extracts above indicate, similar conceptions of dementia are held by patients on the geriatric ward, and when they are offered the cognitive tests some feel that these conceptions become attached to them as persons, an experience that may arouse feelings of fear, anxiety, stress, or sadness (see also Zeiler et al. 2021). Unsurprisingly, the professionals feel a responsibility to meet and try to alleviate these feelings. For this task, however, *tech*nical knowledge about how to apply the written routine is not sufficient. What is also needed is the ability to make careful judgments about what a particular patient, in a particular situation, needs, and a contextually sensitive judgment that puts the professionals into direct contact with a political realm that they feel they have the possibility and responsibility to alter. To paraphrase Arendt, by distancing themselves from their “subjective private conditions” and seeing things from the perspectives of the patients, the professionals become aware of themselves as political actors, as health care workers who act on and within a political realm (Arendt 2006, 217–218). On the one hand, the professionals see it as their responsibility to take political action. As occupational therapist Vera puts it, “it is our responsibility to work with this, around taboos and things”. On the other hand, they see political action as an opportunity that the testing practice itself provides. In doctor Karin’s words, “I think it’s important that we help people with the discomforts, concerns, fears, or prejudices that they can have about certain things, and then this [the testing practice] can be a way of [doing that]”. The professionals feel themselves compelled to exercise a form of political judgment in order to counteract conceptions that exist in the community in which they act.

## The performance and content of Judgment

When the opportunistic screening practice was started, all the occupational therapists and doctors – with the exception of Emelie who was newly graduated – had several years of experience of working in their professional role. They all, however, described undergoing a learning process during the initial phase of the practice. As we saw in the previous section, this involved gaining practical knowledge through working with the tests, which enabled them to make the judgment that the screening practice could be aligned with their profession as a whole. The learning process also made it possible for them to make judgments about how to act in particular test situations, with regard to particular patients. This is how Klara describes this process:

At first, we were probably pretty bound by the criteria [the written routine]. As time has passed, I think we, we’re not completely free to make our own interpretations, but it has become a somewhat different [approach]. You know more about when it’s appropriate and not […]. Now you have a sense of that “this is fair and this isn’t fair” […]. In the beginning we were probably quite hard on ourselves to do it in exactly the same way and to make the same assessments [of the results], and we should still do that and I hope we do, but you hadn’t performed any [tests] yourself then […], when you have done a couple yourself you start to get into it.

Klara’s description of how she and her colleagues went from a primarily rule-based approach to one based on an experiential sense of what is right to do in a particular situation is akin to the process of skill acquisition described by Hubert Dreyfus and Stuart E. Dreyfus (1986) in their book *Mind over Machine: The Power of Human Intuition and Expertise in the Era of the Computer*. An important discovery that Dreyfus and Dreyfus made is that when people meet a practice for the first time, when they are novices, they tend to ask for and hold on tightly to the rules of that practice. But as both they and Klara note, rules can only take us so far in complex practices. As our experience of taking part in a practice grows, we discover the shortcomings of rules and gain practical knowledge that enables us to interpret the situation we are in and make decisions about what is right to do.

Thus, the ethico-political judgment that Klara and her colleagues exercise in the screening practice would not be possible without the experience they gain by taking part in the practice. However, as both Aristotle and Arendt point out, experience does not suffice. We cannot simply rely on our previous experiences when we decide what to do in the current situation. We must view our previous experiences and our virtues in the light of this situation, and *vice versa*. In doing so, we cannot let ourselves be blinded by our prejudices or passions: we must instead apply a reflective attitude towards ourselves, one of disinterestedness, as Arendt would call it, and deliberate about what this situation demands of us. Furthermore, as Arendt’s theory of judgment teaches us, it is because we are subjects who act in a common world that judgment is possible and necessary. It is in this common world that we are accountable for our actions, and it is also because of this common world that we are able to make judgments at all.

“We have had several assessments that we found very difficult,” Liv says, “and you walk away from there feeling ‘Oh, why did I do that test?’.” This short passage tells us a lot. First, it illustrates the responsibility we have for our actions. We are ethico-politically responsible to do what is right, and must be able to justify our actions both to ourselves and to others. Second, it tells us that this is difficult, that there are no guarantees that the action we choose is the right one. Judgment-guided action is always characterized by uncertainty; we can never be sure about the outcome of our actions in advance (Arendt 1998, 178). However, because we live in a world together with others, we have no choice but to act and judge.

This inherent worldliness of our actions and judgments, however, also means that we do not have to regret, question and deliberate about them alone. We can do it together with others. Here, the people together with whom we act and with whom we share our practices may be our most important interlocutors. The interviewees describe that this collective reflection was more intense during the initial phase of the testing practice, but continues today. Sometimes, Liv says, they consult each other about whether to offer the test to a particular patient or not. On other occasions, they discuss how to interpret a patient’s test results. Sometimes the reflection is more formalized and retrospective. This is how Emelie describes it:

We talk a little with each other about different situations sometimes, when you feel that something has been difficult. We have “mirroring” once a week, only among the colleagues […]. Then we can bring up things that we have felt have been difficult in different situations and get some feedback from our colleagues […], “You can think this way or that way or that there is no other way of doing it”.

Clearly, this collective reflection functions both as a way of improving the judgments that are made, by creating a distance to the experienced situations, and as a way of managing the emotional aspects of the testing. As Arendt formulates it, “bound up with judgment is our whole soul apparatus” (Arendt 1992, 74). If our feelings are involved in orienting our judgment, we will also feel the outcome of our actions, which, as Emelie puts it, can be “difficult”. But due to the ongoing collective reflection, Liv feels that she has become “strong in front of the patient”. Now, she says, they all feel strong enough to interrupt the testing if they make the judgment that this is the right thing to do.

Thus, gaining practical knowledge about the testing and engaging in continuous reflection together with one’s colleagues are two essential aspects of what makes judgment possible, and through which it takes shape in the opportunistic screening practice. In the actual test situation, however – where the tests are offered, performed, and evaluated – the occupational therapist is alone with the patient. Here, she must deliberate about what is right to do with regard to a particular patient without the immediate presence of her colleagues. Since judgment “deals with particulars”, as Arendt puts it, there is potentially an inexhaustible number of ways in which this can be done. In our interview material, however, some ways are more salient than others, for example: interrupting the testing, bending the rules of the test instruments by giving patients a bit more time and instruction than allowed, describing and naming the test in different ways, and trying to dedramatize and normalize it. This is, for example, how Klara describes how she sometimes bends the rules,

You have your procedure to follow. So that’s not difficult to know, you know; the order of things is no problem at all. It can be a little difficult when the patients have a lot of questions, but I usually say before the test that “We can discuss how it went afterwards, let’s focus on the test now”. But still there can come a lot of questions or they may seek confirmation. And it can be a little difficult to assess how much confirmation is ok. Because I think, in the test, you should get an instruction once, maybe. Well, but then you also have to have the means to understand an instruction [….]. You [as an occupational therapist] have to assess that in the moment. And it can be a little difficult to know how “kind”, within quotation marks, you should be […]. Because [to seek for] confirmation can also be a health factor with regard to cognitive ability […], but it can also be an insecurity. So sometimes I have deviated from [the test manual] and given repeated instructions.

As Klara’s words indicate, on paper, the test procedure is not difficult at all. The order of things is laid out distinctly in the test manuals. But the procedure can be difficult in reality. The testing practice involves the interaction with a unique individual, whose actions, reactions, and overall situation must be met and dealt with in a particular way. Again, we see that judgment is characterized by difficult deliberations – often with oneself – and uncertain interpretations and outcomes. Yet, it is necessary. In Klara’s view, even though it is difficult to know what to do, how to deal with the patient’s questions and need of confirmation, it would not be right of her to ignore them. She must make an interpretation and assessment there and then about how much she should bend the rules of the test instruments, how much confirmation she should give, and what this tells her about the patient in front of her.

These acknowledgements of the patient’s situation and adaptations of the routine and the test instruments were brought up by some of the patients during the interviews. Klas, for example, made a mistake when performing the clock test. But then they “discussed it a little bit and she [the occupational therapist] questioned me and asked ‘Is this correct?’ And then we realized that ‘No, it isn’t’. How foolish one can be?” In this example, the occupational therapist had realized that Klas had made a mistake unrelated to his cognitive abilities and had therefore made the judgment that it was right to bend the rules a little and discuss the matter with him.

Another way in which the occupational therapists exercise judgment in the testing practice is by adapting their way of informing about and naming the tests for the patient in front of them. Informing about the tests, Vera says, can be particularly challenging if “you’ve heard from the relatives that [the patient] experiences difficulties at home or if you suspect that there are difficulties, […] then it can become more sensitive”. In such situations, the way she describes the tests becomes even more important, she contends. “I try to describe it in a way that the patient won’t think that it’s such a big thing, well, dedramatize it a little”. “There is still a taboo around cognitive impairment”, she says, “so to come to terms with it in your professional role and talk about it in a natural way is pretty challenging I think, absolutely”. One challenge seems to consist in “saying it in an honest way”, as Vera puts it, while being careful not to induce unjustified worry by alluding to the one-sided understanding of cognitive impairment and dementia in the popular discourse.

Informing about the tests is thus far from a mechanical task. As Vera’s words tell us, when offering and performing the tests it is necessary to take into account *both* the patient’s particular situation and condition *and* the sociocultural context in which cognitive impairment and dementia are loaded with preconceptions. In order to prevent the tests being blown out of proportion, the professionals try to dedramatize and normalize them, while still providing a true representation of them. One way of doing so is to use alternative names for the tests.

Liv says that the names she gives to the tests depend on the patient. Most often she avoids the term “cognitive”, since it can be difficult to understand. More often she uses the term “paper-and-pen test”, a term that they have come up with collectively on the ward in order to focus on the concrete contents of the tests and to dedramatize and normalize them. Other names that are used in the interaction with the patients are “memory test” or, more thoroughly, a test that “checks memory, attention, and concentration”. The term no one uses is “dementia”. Doing so would, according to the professionals, both be a misrepresentation of the test – since it is not diagnostic – and induce unjustified worry among the patients.

But no matter how well-deliberated and well-delivered the descriptions and denominations of the tests given by the occupational therapists are, dementia may still enter the room, and with it feelings of sadness, worry, and, on some occasions, deep existential anxiety. Emelie says that she can often tell when a patient makes this association. She describes how, despite her assurance that the test is not a dementia test, the association may linger until the end of the session, when she calculates and delivers the results, and may then give rise to strong emotional reactions. She says:

You have to take your time to meet those reactions also, so that they can let go of it a little, so they don’t get stuck in it, and become extremely anxious about “What has this test said about me?” […] and “What’s going to happen?”, and things like that […]. [In order to] meet the reactions afterwards, it’s good to remain in the room and to be alone with them and let it take a while […]. Not everyone reacts negatively and becomes sad, but it happens, and then it’s good to sit peacefully and calmly and go through it with the patient, give them the opportunity to ask questions and things like that also.

Again, from a *tech*nical viewpoint, things are quite simple; the tests detect cognitive impairment, not dementia, and take around 20 min to perform. From an ethico-political point of view, however, the tests can be delimited neither from the professional ethos that orients the actions of the professionals as a whole nor from the sociocultural context in which dementia is understood as a catastrophic condition and in which cognitive tests are often associated with dementia. In order to act in such a situation, the professional in question must not only know what the right thing to do is, but must also be able to meet the particular concerns expressed by the patient. Judgment is thus a matter of knowledge in the form of *phronesis*, that is, an experiential knowledge that aims to bring to reality the virtues one aims at by constantly renewing and reforming itself in the form of action in a particular situation. As Arendt points out, this requires the combination of a community sense and imagination. In our case, this means that the professionals must imagine what the tests and their results look and feel like from the perspective of the patient, which, in turn, requires a sense of the contextual dimensions that orient this perspective. Here, judgment is not just a matter of deciding the right action in relation to a particular patient: it also involves judging actions within a political realm, where conceptions of dementia and cognitive impairment are held that are deemed to be misleading and detrimental.

## Conclusions

Clinical judgment in screening is an underexplored phenomenon. In this article, we have examined the role and practice of such judgment in a particular form of screening, population-based opportunistic screening for cognitive impairment. On paper, this appears to be a highly formalized and manual-based practice, similar to population-based systematic screening. What we found, however, was that a form of ethico-political judgment plays a decisive role in this practice. Our aim was to gain a deeper understanding of why the professionals deem this form of judgment necessary and how they go about exercising it. We have in the analysis drawn on the ideas of Aristotle, for whom judgment is a form of practical knowledge, *phronesis*, that enables a person to determine what is right in a particular situation, and on those of Hannah Arendt, for whom judgment is political, since it involves judging the particular from the perspective of others in one’s community.

We first of all found that, while many of the professionals were initially hesitant towards integrating the tests into their practice, practical knowledge gained by working with the tests enabled them to make the judgment that they could be integrated into the ethos of their profession. Gaining such practical knowledge also enabled them to distance themselves from the written routine and the test instructions sufficiently to make judgments about *if*, *when*, and *how* the tests should be applied in a particular situation.

Our analysis concludes that clinical judgment in the testing practice is needed for two reasons: the practice involves patients who display unique circumstances, needs, and wishes, *and* it is immersed in two influential contexts: the hospital environment and the sociocultural context. This, we found, requires a combination of an ethical and a political judgment, one that is able to determine what is right to do in relation to a particular patient *and* able to take into account and act on the contextual circumstances that shape the material, organizational and experiential dimensions of the practice. The form and content of this ethico-political judgment differ: they may involve bending the rules of the test instruments, interrupting the testing, describing and naming the tests in different ways, and trying to dedramatize and normalize them.

Our analysis shows that this form of ethico-political judgment is in no way external to the testing practice. When the tests are performed, a unique individual enters the room and so too does a sociocultural context in which cognitive impairment in general, and dementia in particular, are loaded with pervasive preconceptions. Simply following the written routine and the test instructions is not an alternative; one has to let the particularities of the situation in its context shape one’s actions.

Previous research on clinical judgment has focused mainly on its ethical dimension. We have highlighted in this article the ways in which this form of judgment is also political. With the help of Arendt’s theory of judgment, we have shown that judging the particular from the perspective of the other as a member of the community is an essential feature of clinical judgment, since medical practice is not isolated from the world in which it takes place. This should be explored further in future research.

## Footnotes

1 To our knowledge, this is the only hospital in Sweden that has introduced such a program.

2 Screening for dementia is a controversial issue. On the one hand, there is widespread agreement about the benefits of detecting and diagnosing dementia early in the disease process – and screening can lead to early detection (see e.g. Ashford et al. 2006; Banerjee and Wittenberg 2009; Yokomizo et al. 2014). On the other hand, a majority of scholars and national screening committees argue that screening for dementia does not fulfil the generally accepted criteria for screening practices, criteria that were first formulated by Wilson and Jungner (1968) in 1968 and that since have been integrated in various forms into national law in many countries (see e.g. Boustani et al. 2003; Ranson et al. 2018). The three main concerns that are raised against screening for dementia are the lack of evidence of the effectiveness of current treatments, the uncertainty about the benefit-to-harm ratio of the available test instruments, and the lack of knowledge about how a positive test result and subsequent dementia diagnosis may affect the screened subject (see e.g. Burn et al. 2018:2; US Preventative Services Task Force 2020; The UK NSC recommendation on Screening for Dementia 2019). The ambition to improve early detection and diagnosis of dementia is, however, strong, and in the absence of any national population-based systematic screening programs – in which every member of a predefined population is offered testing – variations on this type of screening have emerged. One such example is “population-based opportunistic screening” (Gunnarson et al. 2021). Unlike systematic screening, opportunistic screening offers testing to a predefined population when the opportunity arises, most commonly when members of the population are admitted to hospital for other reasons than those that the screening concerns.

3 Temporary delirium is not uncommon among hospitalized patients and causes similar symptoms as cognitive impairment and dementia.

4 For a more thorough account of how the patients experienced and made sense of the testing practice, see Zeiler et al. 2021.

5 However, research on the practice of screening has uncovered other dimensions of this practice not covered by formal screening criteria such as the barriers to the implementation of screening (Minsky-Kelly et al. 2005) and “the bridging work” performed by profeesionals to facilitate implementation (Timmermans and Buchbinder 2012).

6 Virtues such as courage, temperance, liberty, magnificence, magnanimity, pride, patience, truthfulness, wittiness, friendliness, modesty, righteous indignation.

7 In the Aristotle-inspired literature on clinical judgment and professional judgment in general, lists of virtues of character are often constructed, at which the practitioners of a particular practice should aim (see e.g. Pellegrino and Thomasma 1993; Banks and Gallagher 2009). This is not our ambition in this article. Rather, we aim to gain a deeper understanding of why and how clinical judgment becomes such an integral part of the studied practice. For this, it suffices to remember that judgment and virtues of character are always mutually dependent. This does not mean, however, that the virtues of a particular professional practice are a set of professional skills that have no meaning outside that practice. On the contrary, precisely because they are virtues of character that are exercised and cultivated through action and experience, they cannot be fully separated from the acting *person*, nor from the sociopolitical *context* in which they are exercised. Thus, even though the virtues are professional, they always are and become part of a person’s whole life, including the societal and cultural contexts he or she inhabits (cf. MacIntyre 2011).

8 This is indeed a question of emphasis. For Aristotle, the ethical pursuit of *eudaimonia* is also political, since every individual person’s pursuit of the good life takes place in and is dependent on the city (*polis*), and may, as a consequence, result in the *good* city, which would be even “finer and more godlike”, as Aristotle puts it (Aristotle 2011, 1–3 [1094a-1094b], see also Broadie 2002, 10). For Arendt, too, the ethical and political dimensions of judgment are intimately intertwined. In fact, she understands judgment as that which connects the realm of the ethical – which “concerns the individual in his singularity” (Arendt 2003, 97) – and the political – which concerns “the viewpoint of the community or of the world we live in” (Arendt 2003, 79). In order to decide what is right or wrong to do in a particular situation, we need the discerning and deciding faculty of judgment, which is always political in nature, since it relies on the presence of others – the presence of a community – whose perspectives on the issue at hand we must take into account in order to make a judgment.

9 Arendt was never able to develop her theories on judgment fully. When she died of a heart attack in 1975, the title page of what was going to be the third and final part of her book *Life of the Mind*, which was intended to deal with the faculty of judgment, was found in her typewriter. Despite the absence of this final section, Arendt’s writings on judgment are quite extensive, and since her death many scholars have offered their interpretations of them. Today, there is an intense debate about whether one should understand them as constituting two distinct or one continuous theory about judgment. In the first camp, we find influential scholars such as Beiner (1992), Bernstein (1986), and Maurizio Passerin D’Entreves (2006), for whom Arendt went from understanding judgment as a form of political action, in her early writings, to understanding it as a form of retrospective thought exercised by the spectator, in her later writings. In the second camp, we find Linda M. G. Zerilli (2005) and Shmuel Lederman (2015), for example, in whose view Arendt never claimed that the actor exercises judgment; she ascribed this activity only to the spectator. In Zerilli’s and Lederman’s interpretation, however, the spectator and the actor are not two different persons, but two different ways of relating to the world, which are mutually dependent and deeply intertwined. In the present paper, we follow this second interpretation of Arendt’s writings on judgment.

10 The limited space of this article does not allow a thorough account of Arendt’s reading of Kant and the connections she makes between aesthetic and political judgment.

11 Unlike *phronesis*, where deliberation and action form part of one and the same, continuous movement, political judgment is separated from action. Arendt uses Kant’s metaphor of the spectator to illustrate this point. Whereas, as actors we are directly engaged and invested in what takes place, and therefore unable to sufficiently distance ourselves from it, as spectators, we are able to make judgments, since the subject matter of the judgments plays out before us. However, one should not think of the actor and the spectator as two different persons. The “spectator sits in every actor”, Arendt writes (Arendt 1992:63).

12 The patient sub-project has been approved by the Swedish Research Ethics Board (etikprövningsnämnden, approved, Ref. no. 2019–03034). The sub-study with health care personnel interviews was exempt from review, since it focused on these interviewees’ work and workplace in a way that did not address sensitive personal data (confirmed in a statement from the scientific secretary of the board, 31 October 2017).
